# Embryos derived from small follicles (<8 mm) exhibit delayed blastulation: a retrospective time-lapse analysis

**DOI:** 10.3389/fendo.2026.1980066

**Published:** 2026-10-20

**Authors:** Nuri Peker, Ayşen Yücetürk, Özge Karaosmanoğlu, Burak Elmas, İlke Özer Aslan, Bülent Tıraş

**Affiliations:** 1 Department of Obstetrics and Gynecology, Assisted Reproductive Technologies Unit, Acibadem Maslak Hospital, İstanbul, Türkiye; 2 Department of Obstetrics and Gynecology, Acibadem Mehmet Ali Aydınlar University, School of Medicine, İstanbul, Türkiye

**Keywords:** blastocyst, follicle size, ICSI, morphokinetics, small follicle, time-lapse imaging

## Abstract

**Background:**

Oocytes from small follicles are frequently discarded, although they may represent an additional resource in patients with few available oocytes. This study aimed to evaluate whether blastocysts derived from follicles <8 mm exhibit altered morphokinetic patterns compared with those from larger follicles.

**Methods:**

This retrospective study analyzed 62 blastocysts from 18 women undergoing ICSI: 20 from follicles <8 mm and 42 from follicles ≥11 mm. Morphokinetic parameters (tPNa-tB) were recorded by time-lapse monitoring. As several women contributed to both groups, linear mixed models with a random intercept for patient accounted for clustering. Oocyte- and embryo-level rates were compared using generalized estimating equations with patient as the clustering variable.

**Results:**

No between-group differences were observed for tPNa, tPNf or t2–t8. tSB occurred later in the <8 mm group (median 101.2 vs 95.3 h; p = 0.001), as did tB (108.1 vs 104.3 h; p=0.004). Differences persisted after accounting for within-patient clustering (tSB β = 7.91 h; tB β=8.29 h; both p<0.001). Day-5 blastocyst formation per fertilized oocyte was lower in the <8 mm group (28.8% vs 49.3%; p = 0.040), whereas overall blastocyst formation (38.5% vs 56.5%; p = 0.105) and day-6 rates (9.6% vs 7.2%; p = 0.683) did not differ significantly after accounting for within-patient clustering. Euploidy rates did not differ significantly between blastocysts derived from follicles <8 mm (5/10, 50.0%) and ≥11 mm (7/23, 30.4%; p = 0.433), although the number of biopsied embryos was small. Frozen-thawed single embryo transfer of two euploid blastocysts derived from follicles <8 mm resulted in two live births.

**Conclusions:**

Follicle size is associated with the timing of blastocyst development, with embryos from smaller follicles showing an approximately 8-hour delay in blastulation. Day-6 blastocyst rates and euploidy rates did not differ significantly between groups. These exploratory findings suggest that delayed blastulation alone should not be taken as evidence of impaired developmental potential in embryos derived from small follicles.

## Introduction

Follicle size is an important factor in determining oocyte developmental competence in assisted reproductive technology (ART). Larger follicles are more likely to yield mature oocytes, although this relationship plateaus above a certain diameter. Mohr-Sasson et al. reported that the probability of obtaining a mature oocyte from follicles >13 mm was similar to that from follicles ≥16 mm (1). Similarly, Yang et al. reported higher maturation rates from follicles ≥13 mm (2). On the other hand, smaller follicles are more likely to yield immature oocytes and exhibit reduced developmental potential (3, 4). However, the relationship between follicle size and downstream embryo development remains incompletely understood. This question has direct clinical relevance, particularly for patients with low ovarian reserve. In normal responders, oocytes from dominant follicles are preferentially used, and the behavior of small follicles is of limited practical consequence. In poor responders, however, where few oocytes are available, oocytes from follicles <8 mm may offer an additional opportunity to achieve pregnancy, provided their developmental competence is preserved. Consequently, oocytes originating from small follicles may be overlooked despite their potential contribution to embryo yield.

It remains unclear whether embryos originating from smaller follicles exhibit altered morphokinetic behavior during early cleavage and blastocyst formation when assessed using time-lapse monitoring. Nuclear maturity does not necessarily reflect cytoplasmic competence (5, 6), and because metabolic demand increases markedly at blastulation (7), any difference in developmental kinetics between follicle-size groups would be expected to emerge at this stage rather than during cleavage.

In women with diminished ovarian reserve, poor ovarian response, or advanced reproductive age (8), every mature oocyte may contribute substantially to cumulative live birth potential (9). Therefore, defining the developmental competence of embryos originating from small follicles has direct clinical significance and may help optimize oocyte utilization strategies in IVF laboratories. The aim of the present study was to determine whether blastocysts derived from metaphase II (MII) oocytes retrieved from follicles <8 mm display morphokinetic patterns distinct from those derived from larger follicles (≥11 mm), as assessed by time-lapse monitoring. We hypothesized that, if a shorter period of follicular growth translates into subtle cytoplasmic rather than nuclear deficiencies, any difference would emerge at the blastocyst stage rather than during cleavage. Unlike previous reports (10–12), we examined follicles below 8 mm, isolating a genuinely small follicle population, and examined whether any difference in developmental timing was accompanied by differences in blastocyst formation or euploidy.

## Materials and methods

This retrospective study included patients who underwent *in vitro* fertilization (IVF) treatment between 1 March 2025 and 1 August 2025. The study was approved by the Acıbadem University Institutional Review Board (2026-13/608) and written informed consent was obtained from all participants. Eligibility was restricted to women aged 20–45 years. Consecutive eligible cycles were included, with no selection by age or ovarian response. Couples with azoospermia were excluded. These cycles were drawn from an ongoing prospective cohort of 180 patients in which follicles are aspirated and recorded by diameter category as a part of unit's standard practice, predominantly in patients with diminished ovarian reserve or premature ovarian insufficiency; the present analysis is a retrospective evaluation of the 18 patients in whom time-lapse monitoring was used.

### Controlled ovarian hyperstimulation

A GnRH antagonist protocol was applied to all patients. Treatment was initiated on the 2^nd^ or the 3^rd^ day of the menstrual cycle and patients were examined by transvaginal sonography (TVS) to determine the antral follicle count (AFC). A GnRH agonist trigger was used in patients with polycystic ovaries or serum estradiol >5000 pg/mL; a dual trigger (hCG + triptorelin acetate) was used in the remainder. Oocyte retrieval was performed 36 hours after trigger.

### Oocyte retrieval and embryo follow up

Follicles were aspirated under sedation using a 17-gauge double-lumen needle at a constant aspiration pressure of 120 mmHg. Follicles ≥11 mm were aspirated first into dedicated tubes; the needle was then withdrawn and flushed before aspiration of follicles <8 mm into separate tubes. Oocytes from the two categories were placed in separate chambers of a four-well dish and handled separately thereafter. Follicles of 8–11 mm were aspirated as part of routine care and their oocytes were excluded from all analyses. All cumulus-oocyte complexes from a given patient were denuded in a single session 2–3 hours after retrieval and injected immediately afterwards without interruption between groups, those from follicles ≥11 mm first. Embryos were allocated to the time-lapse incubator immediately after ICSI. Each embryo’s own injection time was recorded by the time-lapse system and used as its time zero. Embryo development was monitored to day 6. As only one time-lapse incubator was available, injected oocytes were allocated immediately after ICSI according to incubator availability and before fertilization or embryo development could be assessed. Thus, allocation was not based on embryo quality, developmental progression, fertilization outcome or blastocyst formation. The statement that 20 blastocysts from follicles <8 mm and 42 blastocysts from follicles ≥11 mm were available for morphokinetic analysis refers only to the final number of embryos that ultimately reached the blastocyst stage within the time-lapse system and could therefore be analyzed. No embryos were selectively transferred into the time lapse incubator after fertilization or during subsequent development.

### Follicle grouping

Follicles were categorized into two groups by diameter: <8 mm (small) and ≥11 mm. Follicle diameter was calculated as the mean of two perpendicular diameters. On the day of trigger, the number of follicles in each size category (<8 mm, 8–11 mm, ≥11 mm) was recorded for each ovary; these follicles were re-measured on the day of retrieval and group assignment was based on this second measurement. The 8-11 mm range was excluded from the analysis. As a secondary consequence, the two analyzed populations were separated by a 3 mm margin, reducing potential misclassification arising from intra- and inter-observer variability of two-dimensional follicle measurement. (13). Most follicles in the larger group were clinically estimated to be ≥13 mm, indicating that they predominantly represented advanced or near-dominant follicles. Individual follicle-oocyte matching was not performed.

The blastocysts in the two groups were compared in terms of time to pronuclei appearance (tPNa), time to pronuclei fading (tPNf), time of cleavage to a two-cell embryo (t2), time of cleavage to a three-cell embryo (t3), time of cleavage to a four-cell embryo (t4), time of cleavage to a five-cell embryo (t5), time of cleavage to an eight-cell embryo (t8), time to start of blastulation (tSB) and time to full blastocyst formation (tB). All analyzed blastocysts originated from metaphase II (MII) oocytes, and no blastocyst development was observed from immature oocytes. Blastocyst quality was assessed according to the Gardner and Schoolcraft grading system based on the degree of blastocyst expansion, inner cell mass (ICM) morphology, and trophectoderm (TE) quality. Blastocysts were categorized and recorded at the time of biopsy or vitrification (14, 15).

### Time-lapse & morphokinetic annotation

Embryos allocated to the time-lapse system were cultured individually in an EmbryoScope+ incubator (Vitrolife, Göteborg, Sweden) in Continuous Single Culture-NX Complete medium (CSCM-NXC; FUJIFILM Irvine Scientific, Santa Ana, CA, USA) under 6% CO_2_ and 5% O_2_ at 37 °C, without medium renewal. All morphokinetic timings were expressed as hours after insemination (t0), with t0 defined as the time of sperm injection. All morphokinetic parameters were annotated by a single experienced embryologist according to established criteria (16).

### Preimplantation genetic testing for aneuploidy

PGT-A was performed in seven patients, in all of whom the indication was a history of recurrent pregnancy loss. Biopsy was carried out on 10 blastocysts derived from follicles <8 mm and 23 derived from follicles ≥11 mm, on day 5 or day 6 according to when each embryo reached the blastocyst stage. Trophectoderm biopsies were analyzed by next-generation sequencing using the Ion ReproSeq PGS Kit (Thermo Fisher Scientific), covering all 24 chromosomes (1-22, X and Y). Embryos were classified as euploid (<20% aneuploid cells), mosaic (20-80%) or aneuploid (>80%) (17). For analysis, mosaic results were grouped with aneuploid embryos, as their number was too small to permit a separate category.

### Statistical analysis

The primary outcomes were tSB and tB; maturation, fertilization and blastocyst formation rates, PGT-A results and clinical outcomes were secondary, exploratory endpoints. No *a priori* power calculation was performed, as the sample size was determined by the number of cycles with time-lapse monitoring. Because both follicle-size groups originated from the same women, baseline characteristics are presented for the cohort as a whole as median (range); morphokinetic parameters are presented as mean ± SD and median (range).

Because multiple oocytes and embryos originated from the same woman, the patient was treated as the unit of clustering in all inferential analyses (18). Morphokinetic parameters were compared using the Mann-Whitney U test and linear mixed models with a random intercept for patient (restricted maximum likelihood, Satterthwaite degrees of freedom); the intraclass correlation coefficient (ICC) quantified between-patient variance. Binary outcomes (MII, fertilization, and day-5, day-6 and total blastocyst formation rates) were analyzed using generalized estimating equations with a binomial distribution, logit link, exchangeable working correlation and robust standard errors. The same approach was used to compare blastocyst formation between culture systems within the ≥11 mm group, and between follicle-size groups among embryos cultured in the time-lapse system.

As sensitivity analyses, the mixed models for tSB and tB were repeated (i) in women contributing blastocysts to both follicle-size groups, with within-patient median differences additionally compared by the Wilcoxon signed-rank test; (ii) with maternal age, male factor infertility and trigger protocol as fixed effects; and (iii) after exclusion of couples with male factor infertility.

A mediation analysis with tSB as mediator of the effect of follicle group on tB was used to localize the developmental step at which the delay arose, rather than to infer an independent mediating mechanism; indirect effects were assessed by 95% bias-corrected bootstrap confidence intervals. As a confirmatory analysis, the tSB-tB interval was compared directly between groups using the Mann-Whitney U test and a linear mixed model.

Mixed models were fitted in jamovi (Version 2.6, GAMLj module); all other analyses, including generalized estimating equations and mediation (Hayes’ PROCESS macro, Model 4, 10–000 bootstrap samples, HC3 standard errors), were performed in IBM SPSS Statistics (Version 28). All tests were two-sided with α = 0.05.

## Results

A total of 18 women undergoing ICSI cycles were included. Baseline demographic and cycle characteristics are summarized in **Table 1**.

**Table 1:** Baseline characteristics of the study cohort (n = 18).

Characteristic	Median (min-max)
Age (years)	31.0 (24-40)
Body mass index (kg/m²)	24.1 (19.7-40.8)
Duration of infertility (years)	3.0 (1-10)
Nulligravid, n (%)	9 (50.0)
Nulliparous, n (%)	15 (83.3)
Total antral follicle count	21.5 (9-80)
Male factor infertility, n (%)	3 (16.7)
GnRH antagonist protocol, n (%)	18 (100)
Duration of stimulation (days)	10.0 (7-13)
Trigger, n (%)	
Dual trigger (GnRH agonist + hCG)	14 (77.8)
GnRH agonist alone	4 (22.2)
Oocytes retrieved	26.5 (7-73)
MII oocytes	21.0 (4-39)

A total of 479 oocytes were retrieved, of which 370 (77.2%) originated from follicles ≥11 mm and 109 (22.8%) from follicles <8 mm; oocyte and embryo outcomes according to follicle size are presented in **Tables 2** and **3** shows the descriptive statistics of the blastocysts.

**Table 2:** Sample distribution according to follicle size groups.

Variables	Follicle size			
	≥11 mm (n, %)	<8 mm (n, %)	OR (95% CI)	p
Oocyte number	370 (77.2%)	109 (22.8%)	–	–
MII oocyte number	275 (74.3%)	56 (51.4%)	0.36 (0.25–0.54)	<0.001
Fertilization rate	207 (75.3%)	52 (92.9%)	4.41 (1.56–12.51)	0.005
Day-5 blastocyst number	102 (49.3%)	15 (28.8%)	0.45 (0.21–0.97)	0.040
Day-6 blastocyst number	15 (7.2%)	5 (9.6%)	1.23 (0.46–3.28)	0.683
Day-5 +Day-6 blastocyst number	117 (56.5%)	20 (38.5%)	0.50 (0.21–1.16)	0.105

Distribution of analyzed variables according to the originating follicle diameter (< 8 mm vs ≥11 mm). Values are presented as number (n) and percentage (%).

Comparisons between follicle-size groups were performed using generalized estimating equations with patient as the clustering variable, a binomial distribution, a logit link and an exchangeable working correlation structure. Odds ratios are expressed for the <8 mm group relative to the ≥11 mm group. Percentages are calculated as follows: MII rate, per total oocytes retrieved; fertilization rate, per MII oocyte; blastocyst rates, per fertilized oocyte.

**Table 3:** Descriptive statistics of all blastocysts included in the study.

Morphokinetic parameter	Mean	SD	Median	Minimum	Maximum
tPNa	17.90	± 1.73	18.00	14.00	21.90
tPNf	23.39	± 2.72	23.05	19.30	31.50
t2	25.74	± 2.76	25.35	21.80	34.00
t3	35.45	± 3.87	35.80	24.70	44.60
t4	37.31	± 3.30	37.25	31.70	46.20
t5	46.95	± 6.29	47.50	33.20	59.80
t8	56.23	± 7.19	56.50	40.60	74.90
tSB	98.88	± 8.57	97.60	84.50	125.80
tB	106.98	± 9.20	105.65	87.80	134.80

Overall descriptive data for morphokinetic parameters of all blastocysts, regardless of follicle size.

* tPNa, time of pronuclei appearance; tPNf, time to pronuclei fading; t2, time of cleavage to a two-cell embryo; t3, time of cleavage to a three-cell embryo; t4, time of cleavage to a four-cell embryo; t5, time of cleavage to a five-cell embryo; t8, time of cleavage to an eight-cell embryo; tSB, time to start of blastulation; tB, time to full blastocysts; SD, Standard deviation.

The MII rate was lower in the <8 mm group (51.4% vs 74.3%; OR 0.36, 95% CI 0.25-0.54; p < 0.001). Among MII oocytes, the fertilization rate was higher in the <8 mm group (92.9% vs 75.3%; OR 4.41, 95% CI 1.56-12.51; p = 0.005); this comparison is restricted to oocytes that had already reached metaphase II, which represent a selected subset of the oocytes retrieved from small follicles. The day-5 blastocyst rate per fertilized oocyte was lower in the <8 mm group (28.8% vs 49.3%; OR 0.45, 95% CI 0.21-0.97; p = 0.040), whereas the day-6 blastocyst rate did not differ (9.6% vs 7.2%; OR 1.23, 95% CI 0.46-3.28; p = 0.683). After accounting for within-patient clustering, the difference in the overall blastocyst formation rate per fertilized oocyte was not statistically significant (38.5% vs 56.5%; OR 0.50, 95% CI 0.21-1.16; p = 0.105). The width of these confidence intervals indicates that the study was not powered to demonstrate equivalence for any of these outcomes.

All embryos derived from follicles <8 mm were cultured in the time-lapse system, whereas embryos from follicles ≥11 mm were distributed between the time-lapse and conventional incubators. Within the ≥11 mm group, the blastocyst rate per injected oocyte was lower in the time-lapse system than in conventional incubators (32.6% vs 50.0%; OR 0.41, 95% CI 0.27-0.63; p < 0.001). When the comparison between follicle-size groups was restricted to embryos cultured in the time-lapse system, blastocyst rates no longer differed (<8 mm 35.7% vs ≥11 mm 32.6%; OR 1.19, 95% CI 0.56-2.54; p = 0.657).

No between-group differences were observed for the early stages (tPNa, tPNf, t2, t3, t4, t5, t8). Median tPNa was 17.9 h (14.0–21.9) in ≥11 mm and 18.0 h (16.9–21.8) in <8 mm (U = 481.5; p=0.354). Similarly, tPNf occurred at 22.9 h (19.3–30.1) vs 23.9 h (19.8–31.5) (U = 471.5; p=0.438). No significant differences were found for 2-cell, 3-cell, 4-cell, 5-cell, or 8-cell stages (all p > 0.05).

tSB was later in <8 mm (median 101.2 h; 90.9–125.8) than ≥11 mm (median 95.3 h; 84.5-110.1) (U = 632.5; p = 0.001). tB was also delayed in the <8 mm group (Median = 108.1; min–max = 99.3-134.8) compared with the ≥11 mm group (Median = 104.3 h; min–max = 87.8-126.1; U = 612.0; p = 0.004). **Table 4** shows the comparison of two groups in terms of morphokinetic parameters.

**Table 4:** Comparison of morphokinetic parameters between follicle size groups.

Variable	Size group	Descriptive statistics					Mann Whitney U	
		M	SD	Med	Min	Max	Test value	P value
tPNa	≥11 mm	17.77	± 1.95	17.90	14.00	21.90	481.5	0.354
	< 8 mm	18.17	± 1.17	18.00	16.90	21.80		
tPNf	≥11 mm	23.25	± 2.72	22.85	19.30	30.10	471.5	0.438
	< 8 mm	23.68	± 2.78	23.85	19.80	31.50		
t2	≥11 mm	25.61	± 2.77	25.05	21.80	32.30	474.0	0.416
	< 8 mm	26.01	± 2.79	26.10	22.20	34.00		
t3	≥11 mm	35.65	± 4.11	35.80	24.70	44.60	403.0	0.798
	< 8 mm	35.04	± 3.38	35.90	27.90	38.80		
t4	≥11 mm	37.10	± 3.58	36.10	31.70	46.20	499.0	0.234
	< 8 mm	37.75	± 2.65	38.00	32.40	42.40		
t5	≥11 mm	47.08	± 6.50	47.55	34.00	59.80	410.0	0.880
	< 8 mm	46.67	± 6.00	47.25	33.20	57.00		
t8	≥11 mm	55.92	± 6.40	54.80	44.90	71.60	443.0	0.729
	< 8 mm	56.90	± 8.77	56.95	40.60	74.90		
tSB	≥11 mm	96.24	± 6.42	95.30	84.50	110.10	632.5	0.001
	< 8 mm	104.42	± 9.95	101.15	90.90	125.80		
tB	≥11 mm	104.23	± 6.88	104.30	87.80	126.10	612.0	0.004
	< 8 mm	112.75	± 10.85	108.05	99.30	134.80		

Comparison of developmental timing parameters between blastocysts derived from follicles < 8 mm and ≥11 mm.

*tPNa, time of pronuclei appearance; tPNf, time to pronuclei fading; t2, time of cleavage to a two-cell embryo; t3, time of cleavage to a three-cell embryo; t4, time of cleavage to a four-cell embryo; t5, time of cleavage to a five-cell embryo; t8, time of cleavage to an eight-cell embryo; tSB, time to start of blastulation; tB, time to full blastocysts; SD, Standard deviation; M, Mean; Med, Median; Mixed-model estimates accounting for within-patient clustering: tSB β = 7.91 h, p < 0.001; tB β = 8.29 h, p < 0.001.

Within-patient clustering was substantial (ICC = 0.343 for tSB and 0.308 for tB), indicating that approximately one-third of the total variance in blastulation timing was attributable to between-patient differences. Time-lapse data were available from 18 patients; 13 contributed embryos to the ≥11 mm group and 12 to the <8 mm group, with 7 contributing to both. After accounting for this clustering in linear mixed models, embryos derived from small follicles still reached the start of blastulation significantly later than those from large follicles (β = 7.91 h, 95% CI 3.84–12.00, t(60) = 3.89, p < 0.001), and full blastocyst formation was likewise delayed (β = 8.29 h, 95% CI 3.80–12.80, t(60) = 3.70, p < 0.001). The magnitude of the delay was essentially unchanged from the unadjusted analyses, confirming that the observed difference was not driven by the unequal contribution of embryos across patients.

In the subgroup of seven women who contributed blastocysts to both follicle-size groups (20 blastocysts from ≥11 mm and 12 from <8 mm follicles), the delay was of similar magnitude (tSB β = 8.72 h, 95% CI 3.71-13.74, p < 0.001; tB β = 10.16 h, 95% CI 4.49-15.84, p < 0.001), and comparison of within-patient median values gave consistent results (tSB, p = 0.031; tB, p = 0.078). The estimated delay was essentially unchanged after adjustment for maternal age, male factor infertility and trigger protocol (tSB β = 7.69 h, 95% CI 4.08-11.31; tB β = 7.98 h, 95% CI 3.86-12.09; both p < 0.001) and after exclusion of the three couples with male factor infertility (tSB β = 8.05 h, 95% CI 3.64-12.45; tB β = 8.76 h, 95% CI 4.12-13.40; both p < 0.001).

tSB and tB were strongly correlated (Spearman’s ρ = 0.898). Mediation analysis is presented in **Figure 1** and **Tables 5** and **6**. Because tB necessarily follows tSB, the near-unity path b (B = 0.993, p < 0.001) reflects the expected propagation of timing between two sequential developmental events. Path a (group → tSB) was significant (B = -8.177, p = 0.002), and the total effect was significant (c = -8.516, p = 0.003), whereas the direct effect was not (c′ = -0.394, p = 0.654). The informative result is this non-significant direct effect: once the onset of blastulation was accounted for, no additional delay accrued through to full blastocyst formation, indicating that embryos from small follicles were delayed specifically at the initiation of blastulation and progressed normally thereafter. Indirect effects were estimated using bias-corrected bootstrap (10–000 resamples). This was confirmed by direct comparison of the interval between tSB and tB, which did not differ between groups (median 8.6 vs 7.9 h; U = 332.5, p = 0.190; mixed-model β = 0.33 h, 95% CI −1.45 to 2.11, p= 0.718).

**Figure 1:**
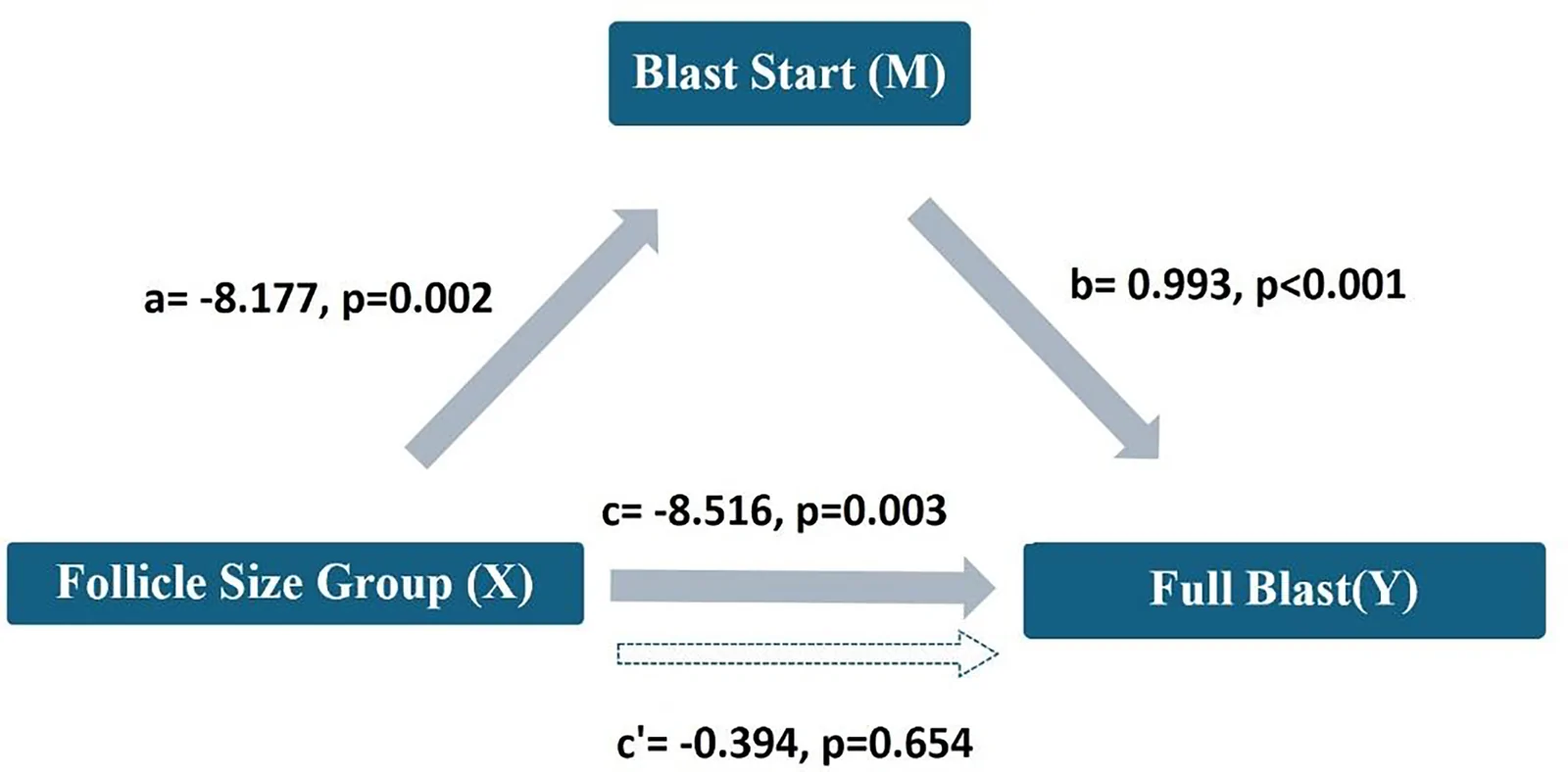
Mediation model diagram. This diagram illustrates the mediation model with Follicle Size Group (X), BLAST START (tSB) (M), and FULL BLAST (tB) (Y). Path coefficients (a, b, c, c′) with corresponding p-values are displayed. C represents the total effect and c′ the direct effect (shown as dashed). Reference group = <8 mm.

**Table 5:** Mediation model coefficients for the effect of follicle size on full blast formation.

Path	B	SE (HC3)	p	95% CI	Standardized β
X → M (a)	-8.177	2.494	0.002	[-13.165, -3.189]	-0.96
M → Y (b)	0.993	0.067	<0.001	[0.859, 1.127]	0.93
X → Y (c’)	-0.394	0.875	0.654	[-2.145, 1.357]	-0.04
X → Y (c)	-8.516	2.712	0.003	[-13.940, -3.093]	-0.93

Regression coefficients for the mediation model examining the indirect effect of follicle size group (X) on full blastocyst timing (Y) through blast initiation (tSB; M).

* X, Follicle size group; Y, Full Blast; M, Blast Start; c, the total effect; c′, the direct effect; HC3, heteroskedasticity-consistent standard errors (type 3); B, Unstandardized regression coefficient; SE, Standard error.

**Table 6:** Indirect effect of follicle size group on FULL BLAST through BLAST START.

Mediator	Indirect effect	Boot SE	95% BC CI	Standardized indirect effect
BLAST START	-8.123	2.514	[-13.239, -3.339]	-0.88

*Indirect effect = -8.123. 95% BC CI [-13.239, -3.339]; because the confidence interval did not include 0, the indirect effect was considered statistically significant (α = 0.05). PROCESS does not report a p-value for indirect effects. These results indicate that the delay in full blastocyst formation was established at the onset of blastulation, with no additional delay thereafter. *Boot SE, bootstrap standard error; BC CI, Bias-corrected confidence interval.

PGT-A was performed in seven patients, all of whom contributed blastocysts to both follicle-size groups. Among the 10 biopsied blastocysts derived from follicles <8 mm, 5 (50.0%) were euploid, 1 was mosaic and 4 were aneuploid. Among the 23 biopsied blastocysts derived from follicles ≥11 mm, 7 (30.4%) were euploid. The difference in euploidy rate between groups was not statistically significant (p = 0.433). This comparison should be interpreted with caution: 14 of the 23 blastocysts from the ≥11 mm group were contributed by a single patient, and when this patient was excluded the euploidy rates were 55.6% (5/9) and 44.4% (4/9) respectively (p = 0.637). The number of biopsied embryos was insufficient to demonstrate equivalence between groups.

When more than one suitable blastocyst was available, embryos derived from follicles ≥11 mm were preferred for transfer. Frozen-thawed single embryo transfers of blastocysts derived from follicles <8 mm were performed in two patients for whom no such alternative was available, and both resulted in a live birth. These outcomes are reported as observations in a very small number of transfers and do not permit inference regarding implantation or live-birth rates.

## Discussion

Embryo assessment is one of the most important steps to enhance clinical pregnancy and live-birth rates in IVF cycles. In the present study, we primarily evaluated whether the diameter of the follicle influences downstream embryo morphokinetics. In this exploratory analysis, early cleavage dynamics were comparable between groups, whereas embryos derived from follicles smaller than 8 mm exhibited a delay at the blastocyst stage. Two live births arising from embryos derived from follicles <8 mm are encouraging observations, but the number of transfers was far too small to support any inference about implantation or live-birth potential. The delay was of similar magnitude after adjustment for maternal age, male factor infertility and trigger protocol, and in the seven women who contributed embryos to both groups, in whom these factors were by definition identical for the two follicle-size categories.

The stage-specific nature of this delay is biologically plausible. All embryos originated from MII oocytes, confirming equivalent nuclear maturity; however, nuclear maturity does not guarantee cytoplasmic competence (5, 6); a distinction also emphasized by Kahraman et al. in the context of follicle size (10). Oocytes from smaller follicles undergo a shorter period of follicular growth, and it has been hypothesized that they accumulate smaller stores of maternal mRNA, proteins and mitochondria and receive less cumulus-mediated metabolic support (5, 6, 19, 20). None of these parameters was measured in the present study, and this explanation therefore remains hypothetical. Early cleavage divisions rely largely on these pre-existing maternal stores and impose modest energetic demands, which may explain the absence of differences from tPNa through t8 (7). Blastulation, by contrast, is the first stage requiring sustained ATP-dependent ion transport for blastocoel formation and expansion (7, 21). Furthermore, developmental speed is not linearly related to viability; both unusually rapid and unusually slow development have been associated with reduced competence (7, 22), suggesting that a moderate delay in blastulation need not indicate impaired potential. A subtle cytoplasmic or mitochondrial deficit could therefore remain undetectable during cleavage and become apparent at blastulation, as observed here.

Two previous studies have examined embryo morphokinetics according to follicle size, both using higher thresholds than ours. Avraham et al. found no morphokinetic difference between embryos from follicles smaller and larger than 10 mm (12), whereas Kahraman et al., applying a 17 mm threshold, reported significantly lower blastocyst formation and top-quality blastocyst rates from smaller follicles, although embryos from smaller follicles reached the blastocyst stage slightly earlier (10). Our results differ from both, showing delayed blastulation in embryos from follicles <8 mm. Rather than a simple contradiction, these divergent findings may reflect the different follicular populations examined: with thresholds of 10–17 mm, the “small” group predominantly comprises intermediate, still-growing follicles, whereas our <8 mm cut-off isolates a genuinely small population largely excluded from, or diluted within, earlier analyses. The relationship between follicle size and developmental timing may therefore be non-linear. The lower maturation rate in our <8 mm group (51.4% vs 74.3%) is consistent with a less advanced follicular population. Studies of other outcomes point in a similar direction. Wirleitner et al. reported comparable blastocyst rates per MII oocyte from 8–12 mm follicles and live births after their transfer (11), and McCulloh et al., who matched each oocyte to its individual follicle, found that follicle size predicted oocyte maturity and blastocyst formation but not blastocyst euploidy (3). Importantly, none of these studies suggests that embryos from smaller follicles should be excluded on the basis of developmental timing alone.

Our comparison group comprised follicles ≥11 mm rather than only those ≥17 mm typically considered fully dominant. However, most follicles in this group were ≥13 mm, and oocyte maturity from follicles ≥13 mm has been shown to be comparable to that from follicles ≥16 mm (1, 2). The ≥11 mm group therefore predominantly represented advanced, functionally dominant follicles, and the contrast with the <8 mm group reflects a genuine comparison between embryos derived from small follicles and those derived from more advanced follicles.

Two additional observations are relevant to whether follicular origin affects developmental timing rather than competence. First, the lower blastocyst rate in the <8 mm group was confined to day 5 (28.8% vs 49.3%), whereas day-6 rates did not differ significantly (9.6% vs 7.2%), a pattern consistent with delayed rather than failed blastulation, although the study was not powered to demonstrate equivalence. Day-6 blastocysts are associated with lower, but still substantial, live-birth rates than day-5 blastocysts, including among euploid embryos (23, 24). In addition, when the comparison was restricted to embryos cultured in the time-lapse system, blastocyst rates did not differ between follicle-size groups. Because time-lapse and conventional incubation supported comparable blastocyst development in a randomized trial (25), the lower rate observed in the time-lapse system here more likely reflects non-random allocation of embryos between systems than an effect of the incubator itself. Second, although the MII rate was lower in the <8 mm group, the fertilization rate among MII oocytes was higher (92.9% vs 75.3%), which may reflect a selection effect whereby an oocyte reaching maturity within a small follicle represents one of the more competent oocytes of that cohort. This contrasts with the lower fertilization rates reported for oocytes from smaller follicles after conventional insemination (4); all oocytes in the present study were fertilized by ICSI. Given the small number of MII oocytes in the <8 mm group, however, sampling variation cannot be excluded.

Time-lapse monitoring provides continuous, non-invasive assessment of embryo development and has been widely investigated for embryo selection (26), although randomized trials have not consistently demonstrated improved clinical pregnancy or live-birth rates (27, 28). Several early morphokinetic parameters including the timing of pronuclear fading, the duration of the second cell cycle, and blastomere movement between the first and second divisions have been associated with implantation and pregnancy outcomes (29–31). Notably, none of these early parameters differed according to follicular origin in our cohort; the only difference we observed was confined to blastulation.

A practical implication concerns embryo selection. Standard morphokinetic selection algorithms are calibrated on timings derived largely from embryos originating from dominant follicles (22), and delayed blastulation has also been proposed as a marker of aneuploidy (32, 33). In this small sample, however, the delay in embryos from follicles <8 mm was not accompanied by a lower euploidy rate. If this delay reflects the follicle of origin rather than impaired potential, such algorithms could systematically deprioritize embryos from small follicles, which is of particular relevance for patients with few available embryos.

This study has several limitations. It was a retrospective, single-center analysis of 18 patients, with no *a priori* power calculation; the study was not powered to demonstrate equivalence, and the absence of a statistically significant difference in day-6 blastocyst rates, overall blastocyst formation or euploidy cannot be taken as evidence that these outcomes are equivalent between groups. The comparison group was defined as ≥11 mm rather than ≥17 mm because follicles are recorded in three diameter categories (<8 mm, 8–11 mm, ≥11 mm) as part of routine practice in our unit, and the inclusion of some 11–13 mm follicles may have introduced heterogeneity. Follicle diameters were recorded categorically rather than as exact per-oocyte measurements, and individual follicle-oocyte matching was not performed. All morphokinetic annotations were made by a single embryologist who was not blinded to the follicle of origin, so inter-observer agreement could not be assessed. Because all embryos from follicles <8 mm were allocated to the time-lapse incubator, culture system was partially associated with follicle group, and the difference in overall blastocyst formation between groups cannot be attributed to follicle size alone; allocation records were available per injected oocyte rather than per zygote, so this comparison could not be restricted to fertilized oocytes.

PGT-A was performed only in the seven patients with a clinical indication for biopsy, and the number of tested embryos was small in both groups; 14 of the 23 blastocysts biopsied in the ≥11 mm group came from a single patient, so the euploidy comparison rests heavily on one individual and should be regarded as descriptive. Embryos derived from follicles ≥11 mm were preferred for transfer when available, so the few transfers of embryos from follicles <8 mm occurred in patients with no alternative; these clinical outcomes are not representative and are too few to determine whether the observed morphokinetic delay influences pregnancy or live-birth rates.

In conclusion, follicle size was associated with the timing of blastocyst development: embryos from follicles <8 mm showed an approximately 8-hour delay in blastulation onset and full blastocyst formation compared with embryos from follicles ≥11 mm, while early cleavage was unaffected. Because full blastocyst formation necessarily follows blastulation onset, the absence of a direct effect in the mediation model indicates that the delay arose specifically at blastulation and did not increase thereafter. Day-6 blastocyst rates and euploidy rates did not differ significantly between groups, and euploid embryos and live births were obtained from embryos derived from small follicles. These exploratory findings suggest that oocytes from follicles <8 mm may represent an additional resource, particularly in patients with low ovarian reserve, and that they should not be disregarded on the basis of delayed developmental timing alone. Confirmation in larger prospective series is required before this can inform clinical practice.

## Data Availability

The raw data supporting the conclusions of this article will be made available by the authors, without undue reservation.
